# ﻿A new species of *Paracortina* from a Vietnamese cave, with remarkable secondary sexual characters in males (Callipodida, Paracortinidae)

**DOI:** 10.3897/zookeys.1149.99651

**Published:** 2023-02-23

**Authors:** Anh D. Nguyen, Pavel Stoev, Lien T. P. Nguyen, Tam T. Vu

**Affiliations:** 1 Institute of Ecology and Biological Resources, Vietnam Academy of Science and Technology, 18, Hoangquocviet Rd., Caugiay District, Hanoi, Vietnam Institute of Ecology and Biological Resources, Vietnam Academy of Science and Technology Hanoi Vietnam; 2 Graduate University of Science and Technology, Vietnam Academy of Science and Technology, 18, Hoangquocviet Rd., Caugiay District, Hanoi, Vietnam Graduate University of Science and Technology, Vietnam Academy of Science and Technology Hanoi Vietnam; 3 National Museum of Natural History, Bulgarian Academy of Sciences, Tsar Osvoboditel Blvd. 1, 1000 Sofia, Bulgaria National Museum of Natural History Sofia Bulgaria

**Keywords:** Biodiversity, cave fauna, northern Vietnam, southern China

## Abstract

A new millipede species, *Paracortinakyrang***sp. nov.**, is described from a cave in Cao Bang Province, northern Vietnam. The new species is diagnosed by having an extraordinarily long projection on the head of males, reduced eyes, a gonocoxite with two processes, a long and slender gonotelopodite with two long, clavate prefemoroidal processes densely covered with long macrosetae apically, and with a distal, reverse, short spine on mesal side, and a rather sinuous distal part of the telopodite. This is the third species of the genus that is known from Vietnam. A brief comparison of some secondary sexual characters is made.

## ﻿Introduction

The millipede order Callipodida is represented in South-east Asia by three extant families – Sinocallipodidae Zhang, 1993, Paracortinidae Wang & Zhang, 1993, and Caspiopetalidae Lohmander, 1931 (Stoev et al. 2008; Enghoff et al. 2015), as well as by the family Burmanopetalidae Stoev, Moritz & Wesener, 2019 known only from Cretaceous amber deposits in Myanmar (Stoev et al. 2019). Of the three extant families, Paracortinidae the most widespread in South-east Asia and is also the most species rich, with 14 species known to date from China and Vietnam (Wang and Zhang 1993; Zhang 1997; Shear 2000; Stoev 2004; Stoev and Geoffroy 2004; Stoev et al. 2008; Liu and Tian 2015; Enghoff et al. 2015). The family comprises two genera, *Angulifemur* Zhang, 1997 and *Paracortina* Wang & Zhang, 1993, and the latter genus is represented by 12 species, while *Angulifemur* has only two species known from caves in Yunnan, southern China. Four out of 12 *Paracortina* species are cave-dwellers and show some cave-adaptations, although no true troglobites are known until present (Stoev and Geoffroy 2004; Liu and Tian 2015). The family will be revised in another study (Akkari et al. in prep.), in which some of species will be redescribed, together with the description of new taxa.

**A list of the hitherto described species of *Paracortina***

1. *P.carinata* Wang & Zhang, 1993 from Shangrila (= Zhongdian) County, Yunnan, China.
2. *P.chinensis* Stoev & Geoffroy, 2004 from Zhenxiong County, Yunnan, China.
3. *P.leptoclada* Wang & Zhang, 1993 from Shangrila County, Yunnan, China.
4. *P.multisegmentata* Stoev & Geoffroy, 2004 from Ngoc-Lac and Loc Thinh, Thanh Hoa, Vietnam.
5. *P.serrata* Wang & Zhang, 1993 from Deqin County, Yunnan, China.
6. *P.stimula* Wang & Zhang, 1993 from Shangrila County, Yunnan, China.
7. *P.thallina* Wang & Zhang, 1993 from Batang County, Sichuan, and Shangrila County, Yunnan, China.
8. *P.viriosa* Wang & Zhang, 1993 from Shangrila County, Yunnan, and Mangkang County, Tibet, China.
9. *P.voluta* Wang & Zhang, 1993 from Yajiang County, Sichuan, China.
10. *P.warreni* (Shear, 2000) from caves at Hong Mat, Hoa Binh, Vietnam.
11. *P.zhangi* Liu & Tian, 2015 from Cave Qiaoxia Dong, Guizhou, southern China.
12. *P.yinae* Liu & Tian, 2015 from Cave in Yanchang Village, Guangxi, southern China.

Here, we describe a new species of *Paracortina* from Ky Rang Cave, Cao Bang Province, Quang Hoa District, Quoc Toan commune, in northern Vietnam. The species is highly adapted to the cave environment and exhibits several somatic characteristics of troglobionts, such as reduced eyes, elongated legs and antennae, and lack of pigmentation on parts of the body.

## ﻿Material and methods

All specimens were hand-collected from Ky Rang Cave, Cao Bang Province, Quang Hoa District, Quoc Toan commune, in northern Vietnam and preserved in 85–90% ethanol. All morphological characters were investigated with an Olympus SZX16 stereomicroscope. Gonopods were dissected for morphological examination and photographed. Colored images were taken using a Nikon SMZ800N microscope and NIS-Element BR v. 5.20.00 and stacked using Helicon Focus v. 7.0. Images were assembled into plates using Photoshop CS6. The terminology follows Stoev and Geoffroy (2004) and Liu and Tian (2015).

Total DNA was extracted using Qiagen Dneasy Blood and Tissue Kits. A 680-bp fragment of the mitochondrial gene, cytochrome c oxidase subunit I (COI), was amplified and sequenced using a pair of universal primers, LCO1490 and HCO2198 (Folmer et al. 1994). Polymerase chain reaction (PCR) conditions for amplification of the COI gene follow those of Nguyen et al. (2019). ExoSap IT was used to successfully purify amplified PCR products, which were then sent for sequencing to the GenLab Company (Hanoi, Vietnam). COI sequences were checked and confirmed using BLASTN 2.6.0+ search (Zhang et al. 2000) and deposited in GenBank with the number accessions OQ281704, OQ281705, and OQ281706.

The holotype, paratypes, and DNA vouchers were preserved in 90% ethanol and deposited at the Institute of Ecology and Biological Resources (IEBR), Hanoi, Vietnam.

Abbreviations: **PT** pleurotergite/s.

## ﻿Results

### ﻿Taxonomy

**Order Callipodida Pocock, 1894**

**Family Paracortinidae Wang & Zhang, 1993**

#### ﻿Genus *Paracortina* Wang & Zhang, 1993

##### ﻿ Paracortina kyrang sp. nov.

Taxon classificationAnimaliaCallipodidaParacortinidae

779EF539-627B-50EC-9FB9-30E0AFFD9B47

https://zoobank.org/22146F0D-BFA6-4F91-84B5-CEB60238183D

**Figure 1.**
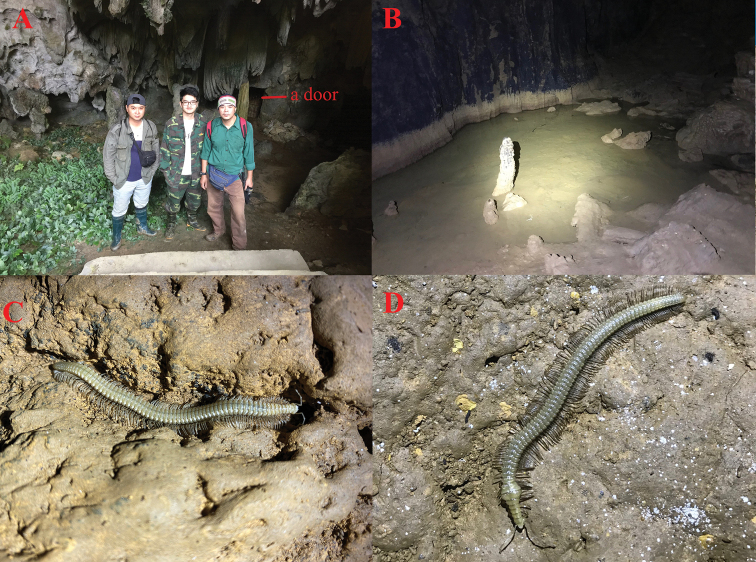
*Paracortinakyrang* sp. nov. **A** entrance of Ky Rang Cave **B** habitat of the species **B, C** habitus, *in situ*. Images not to scale.

**Figure 2.**
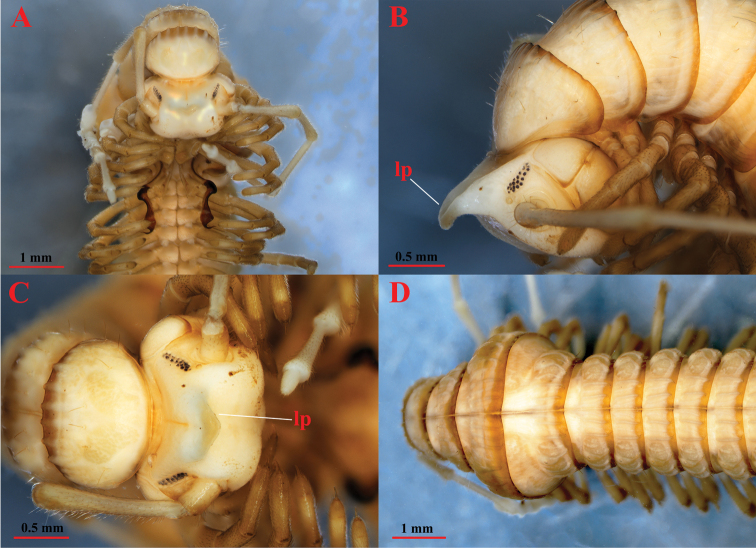
*Paracortinakyrang* sp. nov., holotype **A** anterior body in ventral view **B** anterior body in lateral view **C** head in dorsal view **D** segments 6–12 in dorsal view. Abbreviation: lp = a large projection on head.

**Figure 3.**
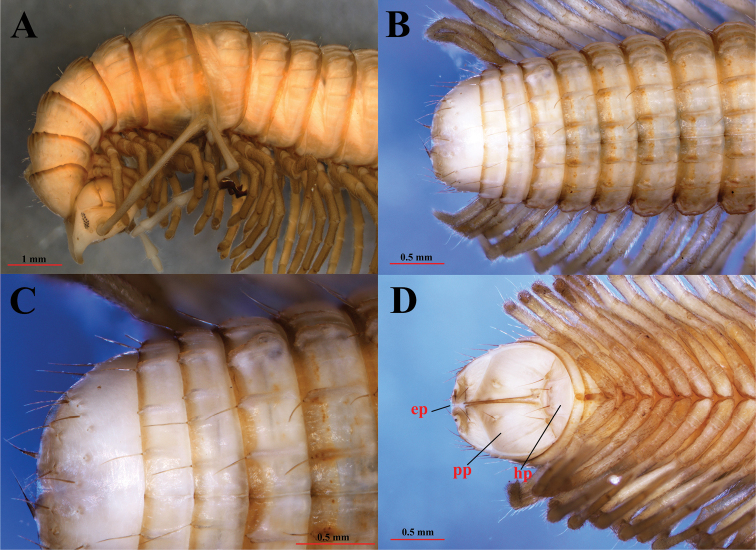
*Paracortinakyrang* sp. nov., holotype **A** anterior part of body in lateral view **B** posterior part of body in dorsal view **C** telson in dorsal view **D** hypoproct, paraprocts, and epiproct in ventral view. Abbreviations: ep = epiproct; pp = paraproct; hp = hypoproct.

**Figure 4.**
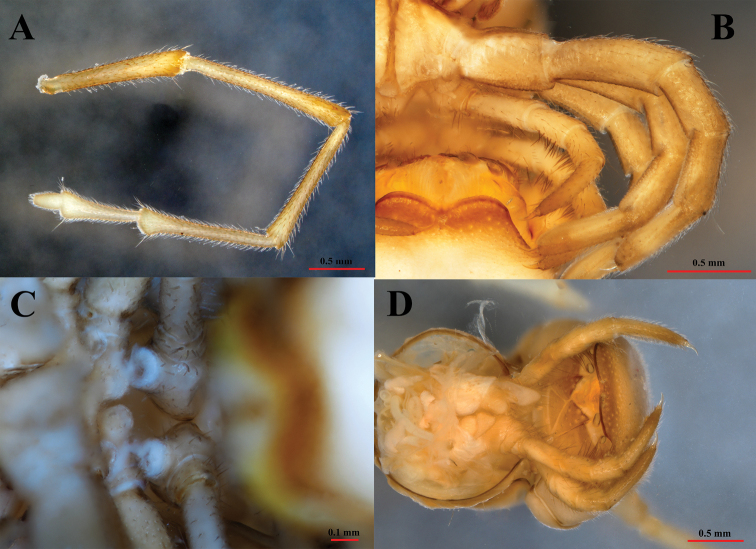
*Paracortinakyrang* sp. nov., holotype **A** right antenna **B** legs 4 and 5 in posterior view **C** gonopores in ventral view **D** female paratype (IEBR-Myr 932), cyphopods in subposterior view.

**Figure 5.**
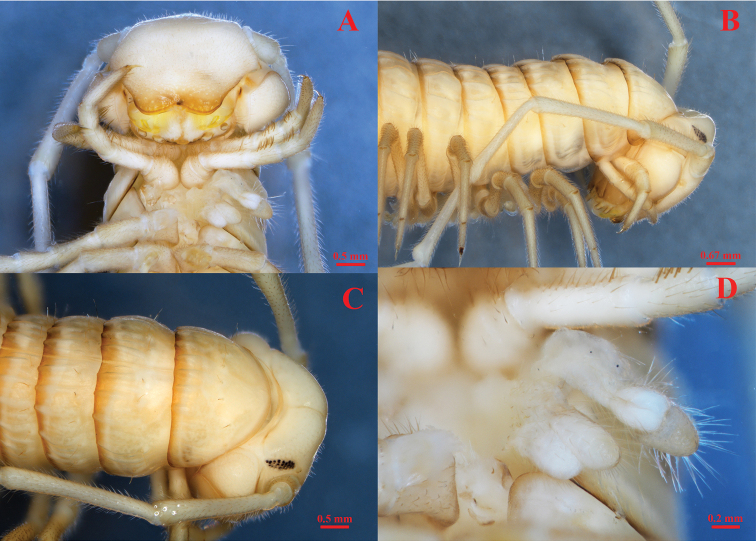
*Paracortinakyrang* sp. nov., female paratype (IEBR-Myr 935) **A** head in ventral view **B** anterior body in lateral view **C** anterior body in subdorsal view **D** cyphopods in ventral view.

**Figure 6.**
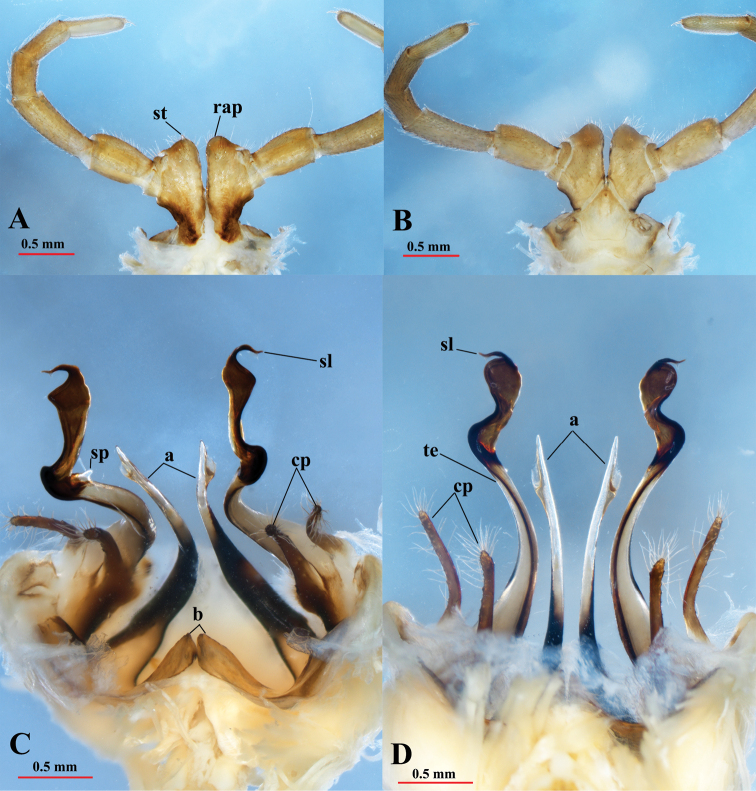
*Paracortinakyrang* sp. nov., holotype **A** leg 7 in posterior view **B** leg 7 in anterior view **C** gonopods in posterior view **D** gonopods in anterior view. Abbreviations: st = short tubercle; rap = rounded anterior process; a = coxal process a; b = coxal process b; te = telopodite; cp = clavate prefemoroidal processes; sp = distomesal spine on telopodite; sl = solenomere.

**Figure 7.**
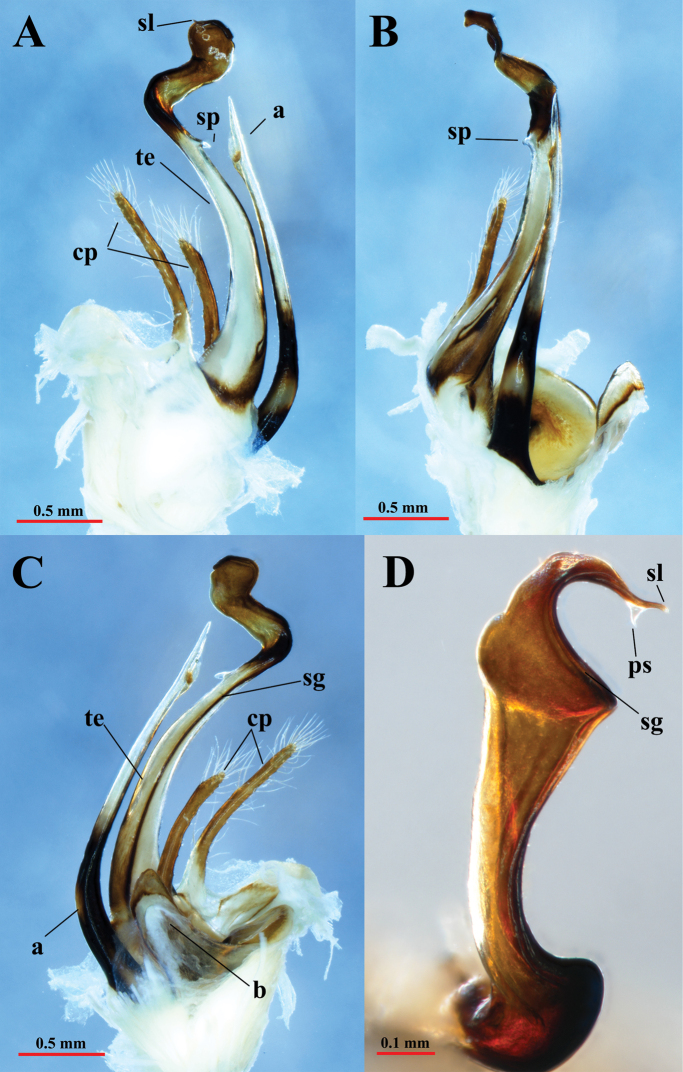
*Paracortinakyrang* sp. nov., holotype **A** right gonopod in lateral view **B** right gonopod in ventral view **C** right gonopod in mesal view **D** solenomere in ventral view. Abbreviations: a = coxal process a; te = telopodite; cp = clavate prefemoroidal processes; sp = distomesal spine on telopodite; sg = seminal groove; sl = solenomere; ps = parasolenomere.

Figs 1 , 2 , 3 , 4 , 5 , 6 , 7

###### Material examined.

***Holotype.*** 1 male (**IEBR-Myr 921**) Cao Bang Province, Quang Hoa District, Quoc Toan commune, Ky Rang Cave, 2.xi.2021, leg. Anh D. Nguyen.

***Paratypes.*** 1 female, 1 juvenile (**IEBR-Myr 932**), 1 female (**IEBR-Myr 935**) same locality, but 17.iii.2022, leg. Anh D. Nguyen & D.D. Nguyen.

***Non-types.*** 1 male, 1 male juvenile, 1 female juvenile (**IEBR-Myr 954**), same locality, but 16.x.2018, leg. Alexandre Faille.

###### Diagnosis.

The new species is well distinguished from all congeners by the strongly modified head in males bearing a unique apically bent projection. Body composed of 68–74 pleurotergites +telson, eyes reduced, composed of 19 or 20 ommatidia in two or three rows. Gonocoxa with an anterior long spiniform process (**a**), as long as ca 80% of telopodite stem, and a rather slender, much shorter, cephalad process (**b**). Process **a** with a cephalad lobe distally, process **b** about 1/3 the length of telopodite. Telopodite with two long, clavate prefemoroidal processes (**cp**), densely covered with long macrosetae apically. Telopodite long, slender, apically twisted laterad, with a distal, reverse, short spine. Distal part of telopodite rather sinuous, narrowed at the base, then smoothly widened at its top, to narrow sharply finally at the solenomere (**sl**) and parasolenomere (**ps**).

The new species can be keyed out into the first branch in Liu et al.’s (2015) key for identification of the species of *Paracortina*, with the clustering species having a pair of prefemoroidal clavate processes (**cp**) on the gonopods: *P.thallina*, *P.stimula*, *P.leptoclada*, *P.voluta*, *P.serrata*, *P.viriosa*, and *P.carinata* (all from southern China).

###### Etymology.

The species epithet “*kyrang*” is a noun in apposition for the type locality, Ky Rang Cave.

###### Description.

***Male holotype***: Length about 42 mm, width and height of midbody PT 2.3 mm and 2.2 mm, respectively; 68 PT+ telson.

***Colour***: living specimens greenish white (Fig. 1C, D). Ethanol preserved specimens: generally white-yellowish; posterior part of metazonites with a brown posterior margin; head, pleurotergites, antennae and telson white-yellowish; legs yellow-brownish.

***Head*** (Figs 2A–C, 3A) highly modified; frons considerably elongated in large projection (**lp**), which is curved at its end; forehead and vertex concave.

***Antennae*** (Figs 2A, 3A, 4B) extremely long, extending beyond the posterior edge of PT 9 when folded backwards; ratio antenna/body length about 1/7; all antennomeres white; length of antennomeres: 1: 0.21 mm, 2: 1.27 mm, 3: 1.26 mm, 4: 1.26 mm, 5: 1.21 mm, 6: 0.67 mm, 7: 0.23 mm; tip of antennomere 7 with four cones protruding well beyond the edge. Eyes (Fig. 2B, C) black, well delineated, composed of 19 ommatidia in three horizontal rows (9+3+7). Tömösváry’s organ about three times larger than the adjacent ommatidium, placed between the eye and the base of antenna (Fig. 2B).

Width of PT: 6>7>>8–14>4>3>2>1. PT slightly broader than high; height of 10^th^PT: 2.19 mm, width 2.32 mm.

***Collum*** (Fig. 2B, C) much narrower than head; pleurotergites 6 and 7 in males strongly enlarged (Figs 2D, 3A). Crests on collum (Fig. 2C) moderately expressed mostly in the posterior part of the segment. Complete crests series appearing from PT2 onwards. Above ozopores, midbody PT with 3+3 primary crests and with 3+3 secondary short crests between primary crests (Figs 2D, 3B); 3^rd^ primary crest strongly enlarged, other primary crests flattened, almost equally broad along the metazonal length; only secondary crests shorter and slightly narrowed posteriorly. Ozopores lying on primary crest 3, visible from sixth to last but two PT (Figs 2D, 3B).

Below ozopores, midbody PT with 2+2 primary crests and 2+2 shorter and thinner secondary crests between primary crests, and 8–10 lower crests down to ventral pleurotergal edge (Fig. 3A).

Midbody pleurotergal setae 5+5, located at caudal edges of primary crests (Figs 2D, 3A); setal pattern as in below (Chaetotaxy). Axial line rather distinct.

***Epiproct*** (**ep**) (Fig. 3B–D) simple, with 7+7 anterior and 4+4 posterior setiferous knobs in transverse rows. Hypoproct (**hp**) (Fig. 4D) tripartite, medial sclerite largest, subtrapeziform, bearing two paramedian macrosetae; each lateral sclerite with a single macroseta born on a large tubercle. Paraprocts (**pp**) (= anal valves) (Fig. 4D) smooth, each divided into a small upper and large lower sclerites, both with a pair of macrosetae. Spinnerets long and slender, ending with a long seta each. All setae on telson brown, contrasting with the yellowish background.

Male leg-pairs 1 and 2 much shorter, with strong setae on ventral side of femorite and tibia, leg-pair 3 slightly shorter than following legs. Tarsi 1–3 1-segmented, and from tarsus 4 to ultimate pair 2-segmented; tarsal pads large until leg 26, then gradually thinner and eventually absent on subsequent legs. All legs ending with a rather slender, long, curved claw. Coxal sacs present from legs 3–26 (PT 16). Only coxae and tibia finely micropapillate ventrally (Fig. 4B).

Coxa 2 with a small anterior process and a posterior gonopore, the latter placed on a small cone (Fig. 4C). Coxa 6 normal, without processes or modifications. Coxa 7 (Fig. 6A, B) with a short tubercle (st), and a very strong, rounded anterior process (**rap**). Coxae of the remaining legs normal.

**Chaetotaxy**:

**Table:** 

	**Anterior setae**	**Posterior setae**
Collum	4+4	2+2
PT2	5+5	broken
PT3	5+5	broken
PT4	broken	5+5
PT5	broken	5+5
PT6-penultimate PT		5+5 (rarely 6+5)

Gonopods (Figs 6C, D, 7) yellow-brownish, some parts dark brown to black (seminal groove, solenomere, basal part of coxal process **a**). Gonopods protruding well beyond the gonocoel, stems of telopodites long, thin, subparallel, and diverging, pointing posteriad (Fig. 6C, D). Coxa with an anterior long spiniform process (**a**), and a rather slender, much shorter, posterior process (**b**) (Fig. 6C). Process **a** as long as ca 80% of telopodite stem, with a lobe distally while process **b** about 1/3 the length of telopodite. Telopodite (te) with two long, clavate prefemoroidal processes (**cp**), densely covered with long macrosetae apically. Telopodite long, slender, apically twisted laterad, with a distomesal, reverse, short spine (**sp**) (Figs 6C, 7A, B). Distal part of telopodite rather sinuous, narrowed at the base, then smoothly widened at its top, to narrow sharply finally at the solenomere (**sl**) and parasolenomere (**ps**) (Fig. 7D). The seminal groove (sg) terminating in the solenomere (Fig. 7D).

**Females.** Head unmodified (Fig. 5A–C). Length about 54.7 mm. The 10^th^PT ca 2.62 mm wide and 2.36 mm high; 68–74 PT + telson. Second leg-pair unmodified. Leg-pairs 1–3 with tarsal brushes. Cyphopods small, densely setose, bilobed (Figs 4D, 5A, D). Coxae 7 normal, without processes.

###### DNA barcode.

The new species has a close genetic identity with *Tetracionjonesi* Hoffman, 1956 (Abacionidae) from 77.74% to 78.25%.

###### Cave habitat.

Ky Rang Cave is located in close proximity to Thang Hen Lake in Cao Bang Province, northern Vietnam, at the altitude of 1,000 m a.s.l. The cave entrance is wide, but the only passage is blocked by an artificial door made by the local residents. Because of it, the semi-light part of the cave is missing, and, on entry, the cave is immediately dark (Fig. 1A). The cave is high (15–20 m), wide (15–20 m), and long (700–1,000 m). The floor is mainly wet with clay and there are some small pools (Fig. 1B). Several other millipede species were found in the cave, for example, *Hylomussrisonchaii* Golovatch, 2019 and *Hyleoglomerisalba* Kuroda, Nguyen & Eguchi, 2022 (Golovatch 2019; Kuroda et al. 2022). The new species was found at a distance of 500 m from the entrance.

## ﻿Discussion

**Figure 8.**
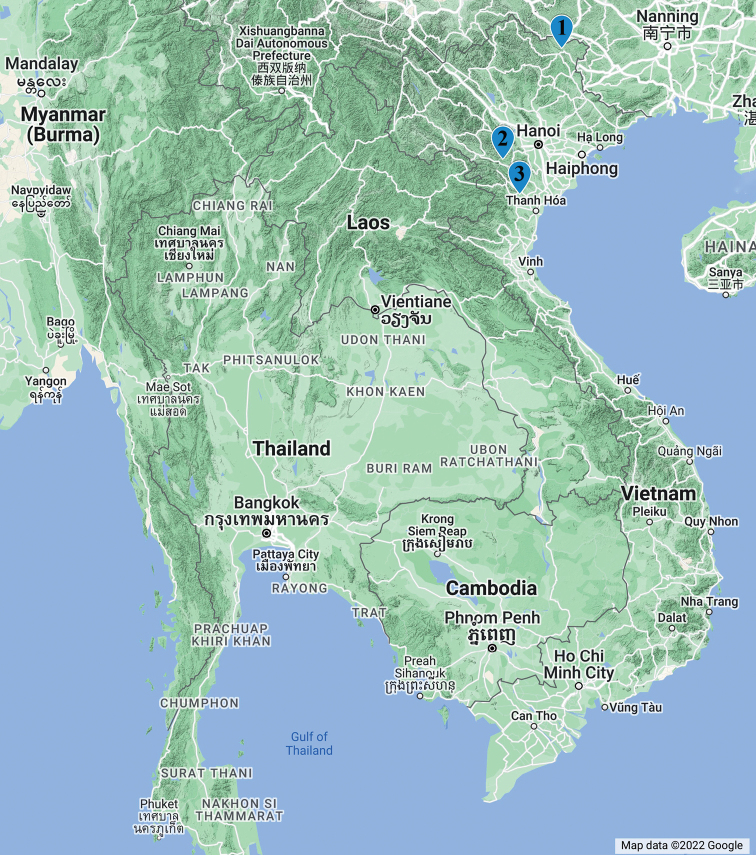
Records of *Paracortina* species in Vietnam **1***Paracortinakyrang* sp. nov. **2***Paracortinawarreni* (Shear, 2000) **3***Paracortinamultisegmentata* Stoev & Geoffroy, 2004.

Currently, there are only three *Paracortina* species recorded in Vietnam: *P.warreni* Shear, 2000 from caves at Hong Mat (Hoa Binh), *P.multisegmentata* Stoev & Geoffroy, 2004 from Ngoc Lac (Thanh Hoa), and *P.kyrang* sp. nov. from Quoc Toan (Cao Bang) (Fig. 8). The first two species have been found on the west side of the Red River, in two nearby localities, while *P.kyrang* sp. nov. is currently known to occur in a single cave on the east side of Red River. This river is known to act as a natural barrier for the distribution of various animal and plant species, including some butterflies (Monastyrskii and Holloway 2013), the spider genus *Nesticella* Lehtinen & Saaristo, 1980 (Ballarin and Li 2018), the frog genus *Microhyla* Tschudi, 1838 (Yuan et al. 2016), gibbons (Hylobatidae) (Geissmann et al. 2000; Thinh et al. 2010), and the plant genus *Cycas* Linnaeus, 1753 (Zheng et al. 2016). Geologically, the northwestern and northeastern Vietnam belong to two different tectonic blocks separated by the Red River. While the northwestern part belongs to the Indochina block, the northeastern part is in the southern boundary of the South China block (Ngo et al. 2014).

Cao Bang Province is located in a karst region of northern Vietnam and supports hundreds of caves varying in size and environmental parameters (Sterling et al. 2006). Recently, several new species have been discovered from caves of Cao Bang Province, including the millipedes *Tylopusnguyeni* Golovatch, 2019, *Parasundaninafaillei* Golovatch, 2019, *Hylomussrisonchaii* Golovatch, 2019, *Hyleoglomerishalang* Kuroda, Eguchi & Nguyen, 2022, and *Hyleoglomerisalba* Kuroda, Nguyen & Eguchi, 2022 (Golovatch 2019; Kuroda et al. 2022), but more new species are expected with more intensive studies and surveys.

Most callipodids live in caves and rock crevices (Enghoff et al. 2015). Completely blind representatives of the order have not yet been found, although some species have reduced eyes, for example, *Sinocallipusjaegeri* Stoev & Enghoff, 2011 from a cave in Laos and *Sinocallipussimplipodicus* Zhang, 1993 from a cave in Yunnan, China (Stoev and Enghoff 2011). Among the members of the family Paracortinidae, *P.warreni* also shows eye reduction, and the species described here also has troglomorphic features.

### ﻿Sexual dimorphic characters in Callipodida

Head shape is often dimorphic in order Callipodida (Ilić et al. 2017). Several species of the families Schizopetalidae, Caspiopetalidae, and Paracortinidae possess modified heads in males, while others, members of Abacionidae, Callipodidae, and Sinocallipodidae, have the conventional convex forehead in both sexes. The head modification can vary form a simple invagination in the forehead area (e.g., representatives of the genera *Acanthopetalum* and *Eurygyrus*), which can sometimes be very pronounced, to triangular protrusions in the middle of the head, such as are observed in most representatives of the genus *Bollmania* (Caspiopetalidae) (Stoev and Enghoff 2005; Enghoff et al. 2015). At least some of the species of Pаracortinidae have a bulge on the head (e.g. *P.zhangi* and *P.yinae*; Liu and Tian 2015), but by no means does *Paracortinakyrang* sp. nov. demonstrate the most extreme case of a projection of the head. The role of these head modifications are not understood but is probably associated with reproduction.

Some callipodidans have the size of the anterior pleurotergites in females and males differing, which allows for observer to determine the sex, even with the naked eye. Usually, in females, the second and third pleurotergites are enlarged, while in males this occurs in the sixth and seventh pleurotergites, where the gonopods are located. The enlargement of pleurotergites in both sexes corresponds to the maturation, when vulvae and gonopods become fully developed. This dimorphic character is observed also in the genus *Paracortina*. The PT 6 and sometimes PT 7 are strongly enlarged in males, but not in females – see *P.chinensis*, *P.multisegmentata* (Stoev and Geoffroy 2004), and *P.kyrang* sp. nov. – and only PT 6 is enlarged in *P.zhangi* and *P.yinae* (Liu and Tian 2015). The enlargement of PT 6 and PT 7 in *P.kyrang* sp. nov. is remarkable, and is not present in other members of the family to the best of our knowledge. In addition, some other characters also differ between males and females: for instance, leg-pairs 1–3 bear tarsal pads in males but tarsal brushes in females; coxa 7 has modified processes in males but is unmodified in females.

## Supplementary Material

XML Treatment for Paracortina kyrang

## Supplementary materials

Supplementary material 1 COI sequences of the new species Data type FAS file https://binary.pensoft.net/file/814467This dataset is made available under the Open Database License (http://opendatacommons.org/licenses/odbl/1.0/). The Open Database License (ODbL) is a license agreement intended to allow users to freely share, modify, and use this Dataset while maintaining this same freedom for others, provided that the original source and author(s) are credited.Anh D. Nguyen, Pavel Stoev, Lien T.P. Nguyen, Tam T. Vu
